# Nasopharyngeal Microbiomes in Donkeys Shedding *Streptococcus equi* Subspecies *equi* in Comparison to Healthy Donkeys

**DOI:** 10.3389/fvets.2021.645627

**Published:** 2021-04-22

**Authors:** Yiping Zhu, Shulei Chen, Ziwen Yi, Reed Holyoak, Tao Wang, Zhaoliang Ding, Jing Li

**Affiliations:** ^1^Equine Clinical Diagnostic Center, College of Veterinary Medicine, China Agricultural University, Beijing, China; ^2^College of Veterinary Medicine, Oklahoma State University, Stillwater, OK, United States; ^3^Dong-E-E-Jiao Co., Ltd., Dong-E County, China

**Keywords:** carrier, donkeys, nasopharyngeal microbiomes, *Streptococcus equi*, 16S rRNA

## Abstract

*Streptococcus equi* subsp. equi (*S. equi*) is the pathogen causing strangles, a highly infectious disease that can affect equids including donkeys of all ages. It can persistently colonize the upper respiratory tract of animals asymptomatically for years, which serves as a source of infection. Several strangles outbreaks have been reported in the donkey industry in China in the last few years and pose a great threat to health, production, and the welfare of donkeys. Nasopharyngeal swab samples for culture and PCR are used widely in strangles diagnosis. Additionally, microbiomes within and on the body are essential to host homoeostasis and health. Therefore, the microbiome of the equid nasopharynx may provide insights into the health of the upper respiratory tract in animals. There has been no study investigating the nasopharyngeal microbiome in healthy donkeys, nor in donkeys shedding *S. equi*. This study aimed to compare nasopharyngeal microbiomes in healthy and carrier donkeys using 16S rRNA gene sequencing. Nasopharyngeal samples were obtained from 16 donkeys recovered from strangles (group S) and 14 healthy donkeys with no history of strangles exposure (group H). Of those sampled, 7 donkeys were determined to be carriers with positive PCR and culture results in group S. In group H, all 14 donkeys were considered free of strangles based on the history of negative exposure, negative results of PCR and culture. Samples from these 21 donkeys were used for microbial analysis. The nasopharyngeal microbiome composition was compared between the two groups. At the phylum level, relative abundance of Proteobacteria was predominantly higher in the *S. equi* carrier donkeys than in healthy donkeys (*P* < 0.01), while Firmicutes and Actinobacteria were significantly less abundant in the *S. equi* carrier donkeys than in healthy donkeys (*P* < 0.05). At the genus level, *Nicoletella* was detected in the upper respiratory tract of donkeys for the first time and dominated in carrier donkeys. It is suspected to suppress other normal flora of URT microbiota including *Streptococcus* spp., *Staphylococcus* spp., and *Corynebacterium* spp. We concluded that the nasopharyngeal microbiome in *S. equi* carrier donkeys still exhibited microbial dysbiosis, which might predispose them to other airway diseases.

## Introduction

*Streptococcus equi* subspecies *equi* (*S. equi*), one of the common upper respiratory tract (URT) pathogens in equids, has been causing the infection referred to as strangles, which still remains an important disease worldwide (1–3). Strangles is highly contagious and pathogenic, resulting in abrupt onset of fever, mucopurulent nasal discharges, coughing and abscesses in submandibular and retropharyngeal lymph nodes (4–6). In cases with severe complications, animals are significantly compromised due to pharynx obstruction, metastatic abscessation (bastard strangles) as well as purpura hemorrhagica (7–9). In uncomplicated cases of high morbidity and low mortality, strangles usually lasts about 25–35 days in most animals (7, 10). However, infection may be extended to chronic or endemic status with constant or intermitted shedding of *S. equi* (7, 11). In most animals, nasal shedding of *S. equi* begins the first few days after onset of pyrexia and persists for 2–3 weeks. However, some animals may keep periodically shedding for a much longer time due to a persistent infection in the guttural pouch (12).

In the last few years, there have been outbreaks of strangles reported in donkeys in China (13, 14). Donkeys <1 year of age were found having much higher morbidity (40.3%) and mortality rates compared to older age groups (13). As the donkey industry has expanded tremendously in China, strangles has emerged as a disease of concern, especially when donkeys are raised at a relatively high stocking density. Additionally, shedding of *S. equi* after the resolution of clinical signs is a potential threat to the industry.

The URT hosts a variety of microorganisms which form a complex microbial community with synergistic and competitive interactions (15). The harmonious coexistence of microbiota is necessary to maintain the health of the URT of animals (16). Dysbiosis of these microbial populations is associated with disruption of respiratory tract health (17) and increased risk of respiratory tract infection (18). The nasopharynx, as part of the URT, frequently harbors both commensal and pathogenic bacteria (19). Investigation of the relationship between nasopharyngeal microbes impacted by respiratory tract diseases is anticipated to increase understanding of the pathogenesis of URT infections (19). Changes of the nasal microbial population before and after long distance transportation in clinically healthy donkeys has recently been reported (17). However, samples obtained using non-guarded swabs might be exposed to contaminants from different parts of the URT including sinuses, nostrils, and oropharynx, each colonized with its own system of microorganisms (20). Therefore, sampling from the deeper nasopharyngeal area may lead to different results.

To our knowledge, there has been no study focusing on the population of donkeys asymptomatically shedding *S. equi*. In this study, we would like to investigate whether donkeys shedding *S. equi* have a perturbed microbial environment in the nasopharynx, which may create a predisposition to other respiratory tract infections (18). Based on this objective, we identified *S. equi* carrier donkeys without signs of clinical disease from a donkey farm that just experienced a strangles outbreak using both culture and PCR techniques. Then nasopharyngeal samples were obtained and the nasopharynx microbiomes in *S. equi* carrier donkeys and healthy donkeys were detected and compared using a 16S rRNA high-throughput sequencing technique. After sequencing, we assessed the differences within the microbial communities between the two groups that were associated with asymptomatic *S. equi* infection.

## Materials and Methods

### Ethics Statement

All procedures involving animals were conducted in compliance and within the license (No.AW11101202-2-0) granted by the Animal Welfare and Ethics Committee of China Agriculture University.

### Animals and Sample Collection

The study was conducted in an intensive donkey farm in Shandong Province with about 800 Dezhou donkeys in total. The recently weaned 6-month-old donkeys were arranged in 24 barns aligned in eight rows with three barns per row. The donkeys were provided with hay and commercial concentrates daily with free choice of water. The farm experienced a strangles outbreak 3 months earlier. It was also the first outbreak known to have occurred in that area. As the commercial strangles vaccine was not available in China, the donkey population on the farm had not been vaccinated against *S. equi* before or after the outbreak. Donkeys affected by strangles was clinically healthy previously according to the health records of the farm. Donkeys which had displayed positive clinical signs were first noted in one barn and quickly isolated. All movement of those donkeys was stopped and a quarantine zone was established to minimize the spread of infection. Their manure and waste feed were also composted separately. A specific group of staff members had been and continued to be assigned to take care of these donkeys using a separate set of equipment, which were disinfected daily. Donkeys in surrounding barns were also limited in movement and monitored closely. Throughout the outbreak, three barns in the same row were affected, while donkeys in other barns remained clinically healthy. Only individuals with severe lymphadenopathy were treated with 3% penicillin gel topically, while other isolated donkeys were monitored three times a day and provided with food and water *ad libitum a*ccording to the local veterinarian's instructions. None of the donkeys involved in this study had been treated with antibiotics throughout the outbreak prior to sampling. The brief signalment and history of the animals involved in this study were summarized in Supplementary Tables 1, 2.

A group of 16 male donkeys of ~6 months of age were randomly selected from the group that had clinically recovered from strangles. Also, none had current clinical signs of disease. These donkeys were designated as group S. Nasopharyngeal swabs were collected using the technique described by Holman (21). Two nasopharynx samples were collected from each donkey. Before sampling, the nostril that was to be used for sampling was wiped clean with 70% ethanol. A 70 cm double-guarded sterile uterine swab (IMV Technologies, L'Aigle, France) was inserted into the nasal cavity passing through ventral meatus gently to approximate depth of 15 cm (22, 23). The guarded inner swab was then extended from the casing, rotated 360° 2–3 times, maintaining contact for about 20 s and then drawn back into the guarded tube. This sample was then stored in a sterile empty vacutainer tube with two to three drops of saline and kept on ice for no more than 6 h. Upon arrival at the laboratory it was used for aerobic culture and PCR. A second sample from the same nostril was obtained using the exact same technique. This swab sample was stored in −80°C until potential 16S rRNA gene sequencing.

A group of 14 male donkeys of approximately 6 months of age was also randomly selected from a different barn located at the furthest row about 200 m away in the same farm, housing donkeys with no history of being exposed and exhibited no clinical signs of *S. equi* infection during the outbreak. These donkeys were still clinically healthy based on a physical exam and were designated as group H. Two nasopharyngeal swab samples were collected with the exact same technique from each donkey, then stored and processed in the same way described above for group S, with the first swab being submitted for aerobic culture and PCR, and the second swab stored for potential 16S rRNA gene sequencing.

### *Streptococcus equi* Aerobic Culture, Biochemical Identification, and PCR

*S. equi* swabs submitted from group S for culture of *S. equi* were processed as previously described (24). Namely, Colombia blood agar (Huankai Microbial, Guangzhou, China) was used to streak the collected swabs for bacterial culture (17). Cultures were incubated at 37°C for 24 h. Isolates were sub-cultured two times on blood agar before being identified. Colonies identified via beta-hemolysis and colony appearance were subsequently tested by Gram staining. Biochemical tests, including ferment lactose, sorbitol and trehalose, were conducted using Micro-Biochemical Identification Tube (Hopebio, Qingdao, China).

DNA from each swab sample was extracted using a Hi-Swab DNA kit (Tiangen, Beijing, China) according to manufacturer's instructions. PCR was performed using primer set of ICESE2GC2F (5′-TTACCTCCATTACTTGACAATCCAT-3′) and ICESE2GC2R (5′-GATTTGCAACATGAAACATTTACAG-3′) (25), which was specific for *S. equi*. Successful amplification was confirmed by 1% agarose gel electrophoresis of the PCR products. Samples with both positive culture and PCR results were selected for further 16S rRNA gene sequencing analysis.

Nasopharyngeal swab samples from group H were tested by aerobic culture and PCR in the same way as described above. Individuals with both negative culture and PCR results were selected for microbial composition analysis with 16S rRNA gene sequencing technique.

### DNA Preparation for 16S rRNA Gene Sequencing

Samples of selected individuals from each group stored at −80°C were thawed for DNA extraction using E.Z.N.A soil DNA Kit (Omega Bio-tek, Norcross, GA, U.S.) in accordance with manufacturer's instructions. Positive DNA amplification was verified by 1% agarose gel electrophoresis. DNA concentration was determined with NanoDrop 2000 UV-vis spectrophotometer (Thermo Scientific, Wilmington, USA).

### High-Throughput Sequencing

The V3–V4 hypervariable regions (26) were amplified by PCR using primer set of 338F (5′-ACTCCTACGGGAGGCAGCAG-3′) and 806R (5′-GGACTACHVGGGTWTCTAAT-3′). The 16S rRNA gene amplification of each sample was performed in a 20 μL reaction system. Reaction conditions were set as follows: initial denaturation at 95°C for 3 min, followed by 27 thermal cycles of denaturing at 95°C for 30 s, annealing at 55°C for 30 s, and extension at 72°C for 45 s. The whole process then ended with a single extension at 72°C for 10 min. 2% agarose gel was used to reveal the PCR products. Extraction and purification of these PCR products were performed using AxyPrep DNA Gel Extraction Kit (Axygen Biosciences, Union City, CA, USA). Amplicons were quantified by Quanti-Fluor^TM^-ST fluorometer (Promega, Madison, WI, USA). Amplicon library pools were gel-sized ahead of sequencing paired-end on Illumina MiSeq platform (Illumina, San Diego, CA, USA).

### Bioinformatics Analysis

FASTQ (v0.20.0) (27) was used to quality-filter the raw 16s rRNA gene sequencing. Sequencing reads were merged using FLASH (v1.2.7). USEARCH (v7.0) was applied to process and cluster sequences for operational taxonomic units (OTU) analysis. OTUs derived from clustering 16S rRNA gene rDNA sequences were used as approximations of microbial taxa (28). The most abundant sequence of each OTU was chosen by QIIME (30) (v1.9.1). RDP Classifier (v2.2) was used to assign a taxonomy with a minimum threshold of 0.7 and the representative sequence of OTU with 97% similarity (29).

### Statistics Analysis

Alpha diversity analysis including Shannon and Chao indexes was carried out using Mothur (v1.30.1) to analyze evenness and richness of OTUs, respectively. Mann–Whitney test was performed to illustrate the diversity difference at different levels between the two groups in this study. The cut-off for rejecting the null hypothesis was set at *P* < 0.05, which indicated no difference between two groups. The OTUs of different levels in each group were assessed by community barplot. Principal coordinate analysis (PCoA) as part of beta diversity was conducted using QIIME (30) (v1.9.1) to reflect the difference and distance between groups based on the Bray–Curtis algorithms. Liner discriminant analysis (LDA) effect size (LEfSe) with an LDA threshold score of >3 was used to determine which taxa and OTUs were most associated with each group and contributed to the differences.

## Results

### Aerobic Culture and PCR Results

Seven donkeys from group S were positive for culture, biochemical identification (non-fermented lactose, sorbitol and trehalose) and PCR. Therefore, they were identified as asymptomatic carriers of *S. equi* and selected for 16S rRNA high-throughput sequencing. In group H, all donkeys remained clinically healthy and yielded negative results on both aerobic culture and PCR. Subsequently, all frozen samples from this group were selected for further microbial composition analysis.

### Microbiota Overview

Nasopharyngeal swabs taken from healthy donkeys and donkeys shedding *S. equi* were assessed by sequencing the bacterial 16S rRNA V3–V4 region. An average of 468 base pairs were obtained from the PCR products. The average DNA concentration yield was 198 ng/μL in group S and 146 ng/μL in group H. A total of 1,385,649 sequence reads were obtained from 21 samples. After removing low-quality reads, singletons, and triplicates, 401,877 sequences were retained (97% sequence similarity). A total of 2,291 OTUs were identified and classified into 36 phyla, 99 classes, 226 orders, 390 families, 835 genera, and 1,326 species. 2,142 and 809 OTUs have been detected in group H and group S, respectively. Raw sequences reads from the samples were deposited at NCBI Sequence Read Archive (SRA) database (accession number PRJNA695404).

### Alpha Diversity Analysis

**Figure 1:**
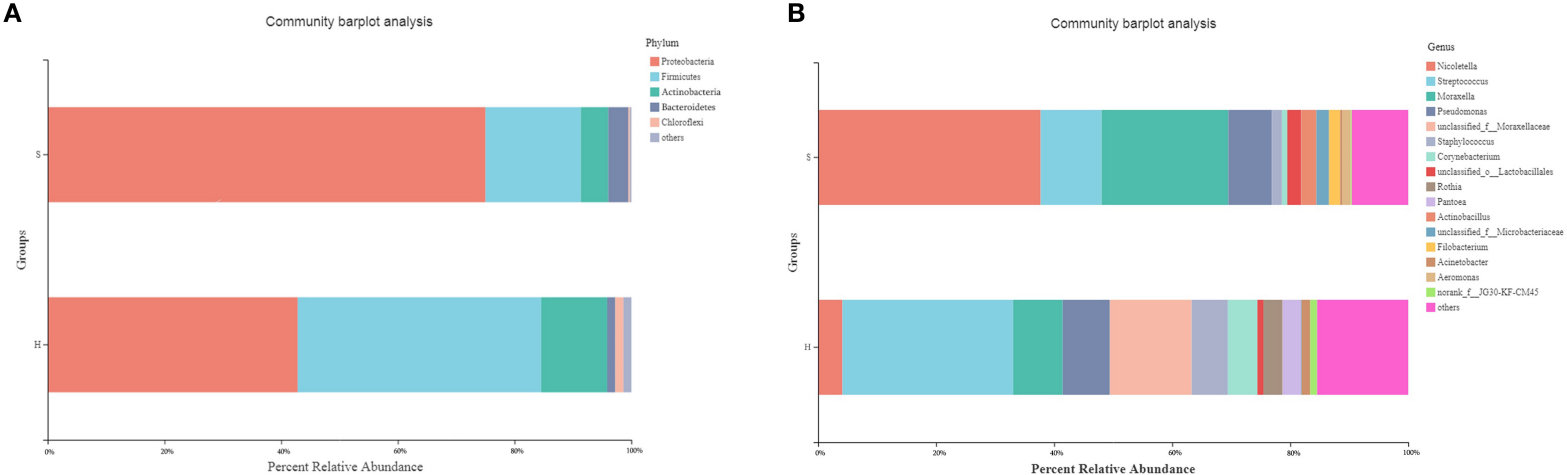
Relative abundance of predominant (>1%) phyla **(A)** and genera **(B)** in the nasopharyngeal microbiota of healthy donkeys and donkeys shedding *S. equi*. H: Healthy donkeys (*n* = 14); S, donkeys shedding *S. equi* (*n* = 7). Other: bacterial taxa with ≤1% abundance, Unclassified, sequences which could not be classified.

Five phyla with relative abundance of >1% were identified in the nasopharyngeal microbiota of the donkeys from group H. The microbial communities of healthy donkeys were dominated by Proteobacteria (42.82%), Firmicutes (41.71%), Actinobacteria (11.32%), Chloroflexi (1.41%), and Bacteroidetes (1.38%) (Figure 1A). There were four phyla with relative abundance of >1% in the communities of *S. equi* carrier (group S) donkeys and they were Proteobacteria (75.01%), Firmicutes (16.32%), Actinobacteria (4.70%), and Bacteroidetes (3.46%) (Figure 1A). In group S donkeys, Proteobacteria were significantly more abundant (*P* < 0.01) than in group H donkeys, while the abundance of Firmicutes and Actinobacteria were notably lower (*P* < 0.05) in group S as compared to group H. At the genus level, 12 genera had a relative abundance of >1% with *Streptococcus* (28.93%) and unclassified *Moraxellaceae* (13.85%) being the most abundant in healthy donkeys. Whereas in group S, 10 genera had a relative abundance of >1% characterized by *Nicoletella* (37.70%) and *Moraxella* (21.48%) (Figure 1B). The relative abundance of *Nicoletella* and *Moraxella* in group S were notably higher (*P* < 0.01) than in group H. While *Streptococcus, Staphylococcus*, and *Corynebacterium* were much lower in abundance (*P* < 0.01) than in group H. There were also a substantial amount of OTUs of unclassified *Moraxellaceae* inhabiting healthy donkeys vs. carrier donkeys.

**Figure 2:**
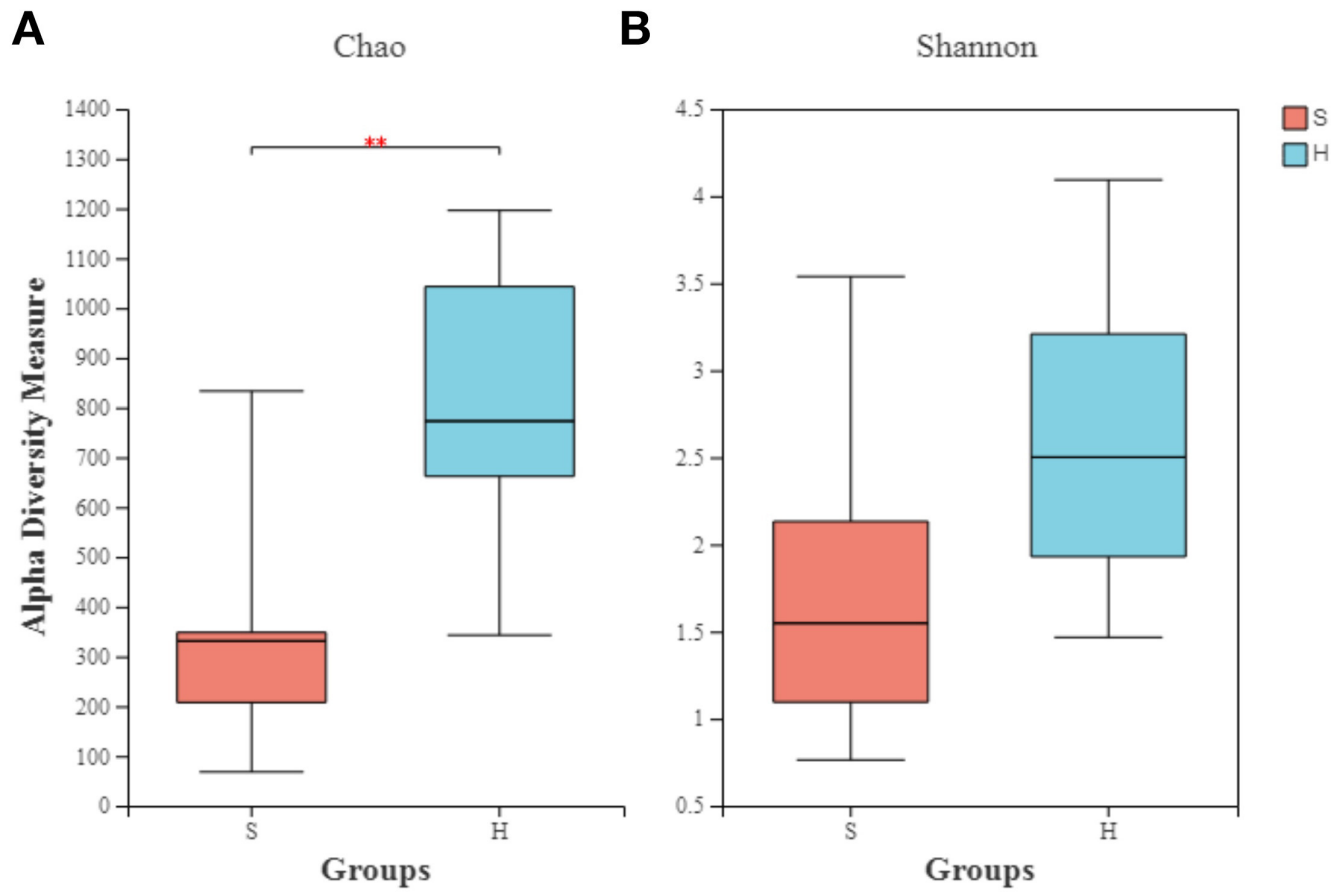
Alpha diversity indexes of the nasopharyngeal microbiota of healthy and carrier donkeys. **(A)** Shannon index of OTU level. **(B)** Chao index of OTU level. S, donkeys shedding *S. equi* (*n* = 7) in orange. H, Healthy donkeys (*n* = 14) in light blue; **significant decrease (*P* = 0.004, Mann–Whitney test) in the richness of the bacterial communities in donkeys shedding *S. euqi* (S) compared to healthy donkeys (H).

The richness (Chao) and diversity (Shannon) of the partitions were calculated. Group S had a microbial composition significantly lower in richness than group H as illustrated by the Chao index (Figure 2A; *P* = 0.004). The Shannon indexes did not differ significantly between the healthy and carrier donkeys (Figure 2B; *P* = 0.057).

### Beta Diversity Analysis

**Figure 3:**
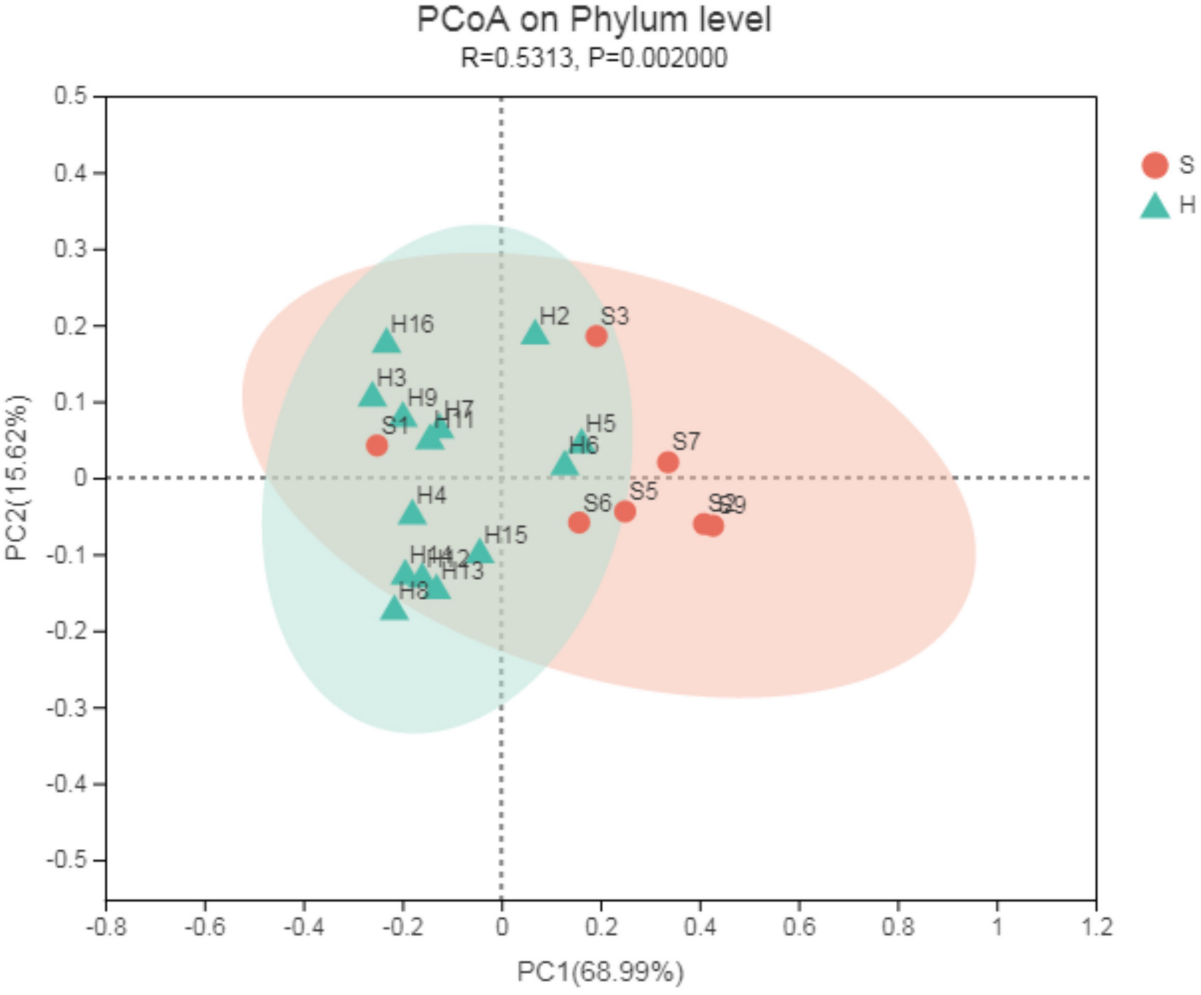
Principal coordinate analysis (PCoA) of the nasal microbiota of healthy and carrier donkeys based on Bray-Curtis distance. S, donkeys shedding *S. equi* (*n* = 7) in orange; H, Healthy donkeys (*n* = 14) in green.

Principal coordinate analysis (PCoA) based on Bray-Curtis algorithms between the two groups was performed and showed differences in microbial communities of the nasopharynx between healthy and carrier donkeys (Figure 3). The ANOSIM of Bray-Curtis distances revealed significant differences of the microbiota composition between group H and group S donkeys (*R* = 0.531, *P* = 0.002).

**Figure 4:**
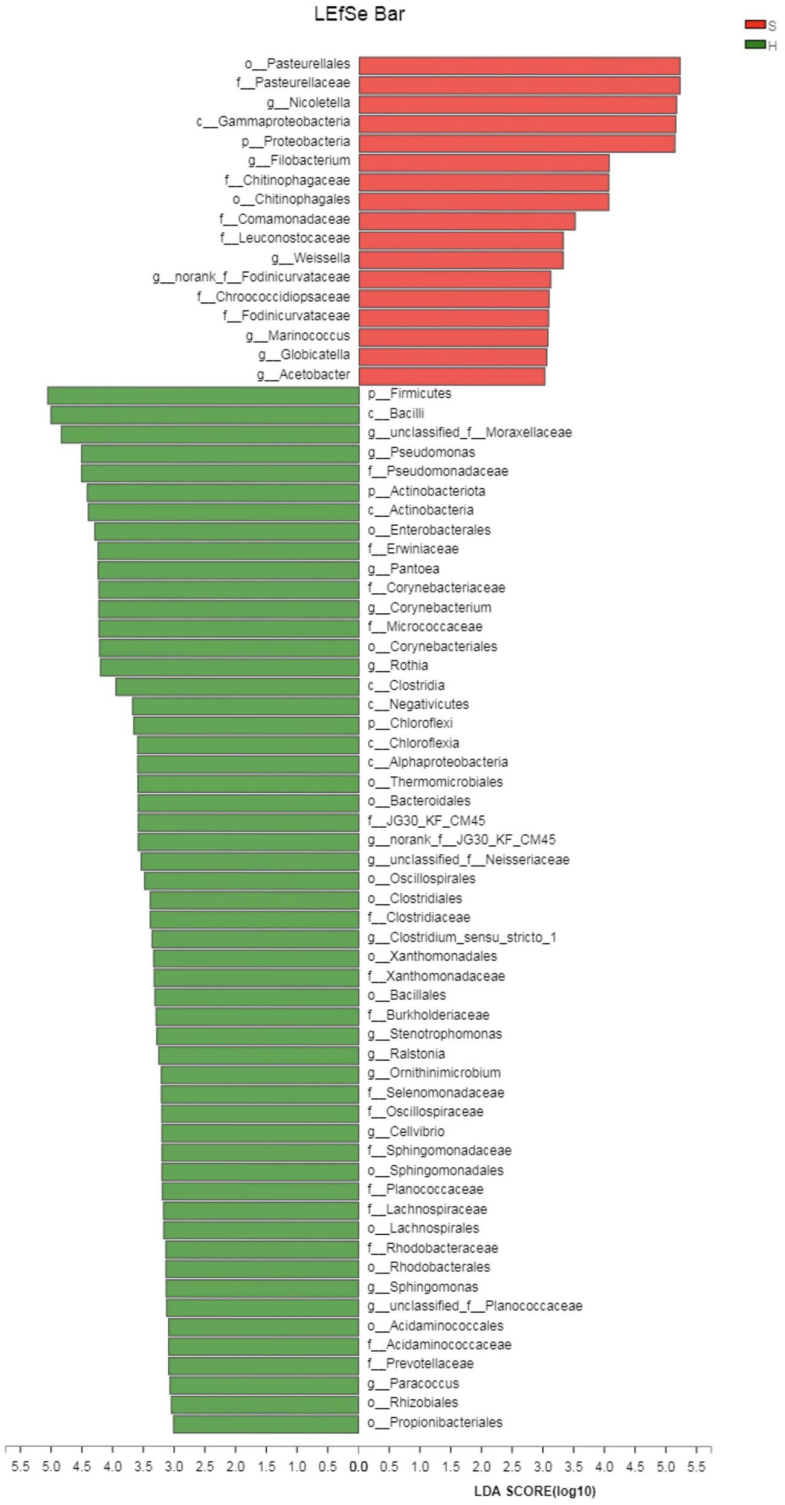
Linear discriminant effect size analysis (LEfSe) of the nasopharygeal microbiota of healthy and donkeys shedding *S. equi*. Bacterial taxa at the genus level and higher in group S (carrier donkeys, in red) and group H (healthy donkeys, in green) were demonstrated by LDA scores >3. Ranking of taxa was based on effect size in LEfSe.

The linear discriminant analysis (LDA) effect size (LEfSe) method was applied to identify high-dimensional biomarkers and assess the differences between the two groups at the genus or higher taxonomic level. The LDA was set to 4.0 as a threshold for identification of biomarkers of different taxa in LEfSe analyses. From all 2,291 OTUs, 257 OTUs were significantly different between the healthy and carrier donkeys (*P* < 0.05). From 835 genera or taxa at higher levels, 379 taxa were significantly different between the two groups (*P* < 0.05); Among them, 70 taxa had LDA scores above 3. Sixteen taxa were associated with group S donkeys and the rest of the taxa (54) were associated with healthy donkeys. The most discriminating taxa in the samples of group S donkeys were Pasteurellales, *Pasteurellaceae*, and *Nicoletella*. *Nicoletella* belongs to the order Pasteurellales and the family *Pasteurellaceae*. In group H donkeys, the most associated taxa were Firmicutes, Bacilli and unclassified *Moraxellaceae* (Figure 4). *Bacilli* is at the class level belonging to the phylum Firmicutes, while *Moraxellaceae* is at the family level belonging to the phylum Proteobacteria.

## Discussion

The previous “gold standard” for detection of *S. equi* was aerobic culture of samples from the upper respiratory tract, including nasal washes, nasal/nasopharyngeal swabs, nasopharyngeal washes or purulent material aspirated from abscesses (31). However, these samples may result in false negatives, especially in the early febrile state. Hence, culture of these samples is no longer considered as the gold standard for strangles diagnosis, even though they are still commonly used (6, 7, 32). Animals recovered from strangles can be asymptomatic carriers and intermittently shed the pathogen (33, 34). They can be a source of infection leading to new or recurrent diseases even in well-managed groups of animals (1) and are also challenging for accurate diagnosis due to the periodical shedding. There is evidence that PCR is more specific and sensitive for detecting *S. equi* than culture (24, 32). Quantitative PCR (qPCR) also referred as real-time PCR has been developed for more sensitive and rapid diagnosis of strangles (35, 36) and guttural pouch lavage qPCR is recommended to detect carriers (6). However, the PCR technique is not able to differentiate viable from non-viable organisms, and it is currently not widely available in China. As a result, a combination of culture and regular PCR was applied to achieve a more reliable diagnosis for carrier donkeys in this study. Since *S. equi* may periodically shed from the guttural pouch into the nasopharynx, rostral nasal swabs may present false negative results (6). However, compared to guttural pouch lavage, swab samples are easier to obtain without the requirement of endoscopic equipment. Hence, we chose to sample the nasopharyngeal area with double-guarded uterine swabs. In this study, 7 out of 16 donkeys from group S were positive on culture and PCR tests. Therefore, they were determined to be asymptomatic carriers. The remaining nine donkeys in group S were not double positive on these tests, therefore, their carrier state was not confirmed at that point and they were not included in this study. Guttural pouch lavage PCR with visual inspection of the guttural pouch guided by endoscopy would be necessary to detect other *S. equi* carriers in this group. The “carriers” defined in the study, along with the other donkeys in the same barn, continued to be isolated, waiting for the future inspection of the local veterinary officials. As for group H, these donkeys had no exposure to *S. equi* and a history free of clinical signs during the outbreak. In addition, they were all under strict biosecurity and negative on both culture and PCR. Thus, they were considered not infected with *S. equi* at that point and were used as negative controls of group S. Ideally, two blood samples for serology test with 2 weeks apart are recommended to help confirm their negative infection status (6).

We hypothesized that asymptomatic carriers of *S. equi* would have dysbiosis of the microbial community of their upper respiratory tract despite absence of clinical signs of disease. The 16S rRNA sequencing results revealed differences between the two study groups and were able to detect microbial changes within carrier donkeys compared to those that remained healthy. Nasopharyngeal samples have been used as diagnostic samples of *S. equi* (32) and representative samples of upper respiratory tract microbiomes (37). To our knowledge the current study is the first to compare the nasopharyngeal microbiota of healthy donkeys with donkeys shedding *S. equi* using high-throughput 16S rRNA gene sequencing.

In this study, the nasopharynx in healthy donkeys hosted richer microbiomes than donkeys shedding *S. equi*. A healthy microbiome is usually characterized by a diverse community of organisms, which are more stable and more resistant to overgrowth of pathogenic microbiomes. Whereas a dysbiotic community, associated with chronic disease, usually has a markedly lower diversity and is often dominated by a few pathogenic species (38). There is also strong evidence that dysbiotic microbial communities are more susceptible to inflammation and diseases (39). The significantly decreased diversity of microbial composition and number of OTUs in group S were consistent with dysbiotic communities within the URT. Similar results were also noted in published research of other species. For example, cattle with bovine respiratory disease complex (BRDC) tend to not only have a less rich source of bacteria, but also lower numbers of OTUs in the nasopharynx, as compared to healthy cattle (21). In humans it has been reported that chronic inflammatory diseases have been associated with a decrease in microbial diversity, evenness, as well as richness (20, 40, 41). This change in microbial richness under circumstances characterized with infection or inflammation was probably due to an increased colonization of anaerobic bacteria, which was facilitated by biofilm formation (42, 43).

According to the microbial composition analysis, the dominant phyla were Proteobacteria, Firmicutes, Actinobacteria and Bacteroidetes in both groups. The results of healthy donkeys in this study are comparable with Zhao's study conducted in clinically healthy donkeys with non-guarded nasal swabs (17). Similar results have also been mentioned in studies of healthy URT microbiomes in horses (37), cattle (21), humans (44), as well as in swine (45). Proteobacteria occupied a notably larger portion of the nasopharyngeal microbiome in donkeys shedding *S. equi* than in healthy donkeys. There is evidence that Proteobacteria is associated with increased severity of inflammation and respiratory tract disease (46). *Moraxella* and *Nicoletella*, the two bacterial genera belonging to Proteobacteria phylum, were predominant in *S. equi* carrier donkeys. *Moraxella spp*. have been considered as pro-inflammatory bacteria associated with asthma in humans and horses (37, 47). It has also been found having a dramatically high relative abundance in cattle with BRDC vs. healthy cattle, which could potentially cause disorders such as pneumonia and otitis (48). In our study, the abundance of *Moraxella* spp. increased significantly in carrier donkeys as opposed to donkeys free of strangles. This is consistent with previous findings in respiratory diseases in other species. Even though donkeys shedding *S. equi* are usually asymptomatic, they are still likely to undergo inflammatory processes in the upper respiratory tract especially guttural pouches (49). In consequence, donkeys shedding S. equi were still having microbial perturbation consistent with the shifted predominance of *Moraxella* in this study.

In donkeys, *Nicoletella spp*. were first identified and reported in our study with a predominant presence in carrier donkeys shedding *S. equi*. *Nicoletella* spp. are rarely reported in other species, except for horses. *Nicoletella semolina* is the species repeatedly isolated from horses with airway disease and first reported in 2004 (50). As a new member in the *Pasteurellaceae* family, it is usually present as part of the normal flora in the equine airway. It has been isolated from URT of healthy horses and horses with airway disease in similar proportions in Europe, however, marked growth of *N*. *semolina* was observed in tracheal aspirate cultures from horses with respiratory disorders (51). Pulmonary disease potentially associated with *N*. *semolina* infection has been reported in three young horses in North America (52). All three cases were characterized with chronic airway infection and heavy growth of *N*. *semolina* was observed in transtracheal wash cultures along with other equine airway flora. The results indicated that *N*. *semolina* might overgrow and cause airway disease in a dysbiotic community. In 2020, *Nicoletella semolina* was first reported as a sole isolate in a horse with a pulmonary infection in New Zealand. Nonetheless, whether it acted as a primary or opportunistic pathogen was still not determined (53). According to results of LEfSe herein, *Nicoletella* was also the genus most often associated with *S. equi* carrier donkeys, while it was not discriminative in the group of healthy donkeys. Therefore, the overgrowth of *Nicoletella spp*. in carrier donkeys might be associated with URT dysbiosis and possible chronic airway inflammation caused by *S. equi*. Further investigation involving more samples will be required to facilitate the understanding of its role in URT diseases in donkeys.

*Streptococcus* belonging to the Firmicutes phylum was another genus showing a significant difference between the two groups at the genus level. Its relative abundance was notably lower in group S donkeys compared to healthy donkeys. Since the classifier used in this study was the RDP Classifier, which is only able to classify 16S rRNA genes from phylum to genus (54), the species of *Streptococcus* were not determined definitively. Our hypothesis to explain this finding was that the predominant *Streptococcus spp*. in healthy donkeys could be *S. equi* subsp. *zooepidimicus*, a common flora in the URT, while in carrier donkeys, the presence of *Nicoletella semolina* might inhibit the normal growth of *S. equi* subsp. *zooepidimicus* and contribute to microbial dysbiosis. Besides *Streptococcus* spp., *Staphylococcus* spp., and *Corynebacterium* spp., which are considered part of microbiota of healthy equine airways (55), also demonstrated significantly lower abundance in Group S. Herein, we inferred that suppression from *Nicoletella semolina* might also lead to this finding.

LEfSe analysis was conducted to identify the most discriminating bacterial taxa of each group. Pasteurellales, *Pasteurellaceae* and *Nicoletella* were the top three taxa associated with donkeys shedding *S. equi*. Thereinto *Nicoletella* belongs to the order Pasteurellales and the family *Pasteurellaceae*. As these bacterial groups were shown at multiple taxonomic levels, the association between these bacterial taxa and donkeys shedding *S. equi* was more robust. Based on LDA, majority of taxa with an LDA score of >3 was associated with healthy donkeys. Hence, there seemed to be no single bacterial taxon strongly associated with healthy donkeys, which reflects a more balanced microbial composition.

Even though strangles has been known to produce chronic shedding individuals, the long-term impact of *S. equi* on the URT from the point of view of microbiomes was still unclear. This study demonstrated a characteristic nasopharyngeal microbiota profile of donkeys shedding *S. equi* and of healthy donkeys, which may promote future research on the microbial dysbiosis of URT in donkeys and other susceptible animals. However, the sample size was relatively small and one round of sequencing analysis only represented the nasopharyngeal microbiota of the point when the samples were taken. Although it would be difficult to perform, larger groups of samples acquired at different stages of the disease may facilitate greater understanding of the pathogenesis, as well as the long-term influence of strangles on the URT of infected animals.

## Conclusions

There were significant differences shown in microbiota analysis between the two groups, including OTU richness and microbial composition at different taxonomic levels. The significantly increased abundance of *Nicoletella* spp. and *Moraxella* spp. in *S.equi* carrier donkeys indicated a dysbiotic URT microbial community possibly associated with chronic airway perturbation. Significantly decreased abundance of *Streptococcus* spp., *Staphylococcus* spp., and *Corynebacterium* spp. in group S was probably due to inhibition by *Nicoletella* spp. Therefore, the role of *Nicoletella* in the URT of donkeys requires further investigation.

## Data Availability Statement

The original contributions presented in the study are publicly available. This data can be found here: NCBI PRJNA695404/SRP304294.

## Ethics Statement

The animal study was reviewed and approved by Animal Welfare and Ethics Committee of China Agriculture University. Written informed consent was obtained from the owners for the participation of their animals in this study.

## Author Contributions

JL and YZ conceived the study. YZ, SC, ZY, TW, ZD, and JL participated in animal acquisition and sample collection. SC helped conduct data analysis. YZ, RH, and SC contributed to the writing of the original draft. RH and JL helped review and edit the manuscript. All authors contributed to the article and approved the submitted version.

## Conflict of Interest

TW and ZD were employed by the company Dong-E-E-Jiao Co., Ltd.

The remaining authors declare that the research was conducted in the absence of any commercial or financial relationships that could be construed as a potential conflict of interest.
