# Supplementary material for: Nasopharyngeal Microbiomes in Donkeys Shedding Streptococcus equi Subspecies equi in Comparison to Healthy Donkeys
Source: Front Vet Sci. 2021 Apr 22;8:645627. doi: 10.3389/fvets.2021.645627 (PMC8100518; doi:10.3389/fvets.2021.645627)
Supplement: Supplementary file 1 [file Data_Sheet_1.docx]

| ID | Group | Sex（Male/Female） | Age (Months） | Symptoms | History of antibiotics |
| --- | --- | --- | --- | --- | --- |
| 9588 | H1 | M | 6 | None | None |
| 1137 | H2 | M | 6 | None | None |
| 1042 | H3 | M | 6 | None | None |
| 3587 | H4 | M | 6 | None | None |
| 4521 | H5 | M | 6 | None | None |
| 4644 | H6 | M | 6 | None | None |
| 2766 | H7 | M | 6 | None | None |
| 3844 | H8 | M | 6 | None | None |
| 5439 | H9 | M | 6 | None | None |
| 1167 | H10 | M | 6 | None | None |
| 3088 | H11 | M | 6 | None | None |
| 2325 | H12 | M | 6 | None | None |
| 1046 | H13 | M | 6 | None | None |
| 2719 | H14 | M | 6 | None | None |

**Table S1. The animal information and brief medical history of Group H.**

**Table S2. The animal information and brief medical history of Group S.**

| ID | Sex（Male/Female） | Age  (Months） | Initial clinical signs observed | | Recovery (Date) | History of antibiotics |
| --- | --- | --- | --- | --- | --- | --- |
|  |  |  | Symptoms | Date |  |  |
| 11AA | M | 6 | Anorexia, lethargy | 2020.7.12 | 2020.8.8 | None |
| 1B93 | M | 6 | nasal discharge | 2020.7.13 | 2020.8.15 | None |
| 9A55 | M | 6 | Submandibular lymphadenopathy | 2020.7.14 | 2020.8.30 | None |
| 3F85 | M | 6 | Nasal discharge | 2020.7.14 | 2020.8.18 | None |
| 1B62 | M | 6 | Anorexia, lethargy | 2020.7.14 | 2020.8.4 | None |
| 4B89 | M | 6 | Anorexia, lethargy | 2020.7.15 | 2020.8.12 | None |
| 7D37 | M | 6 | Anorexia, nasal discharge | 2020.7.15 | 2020.8.8 | None |
